# Efficient lytic induction of kaposi's sarcoma-associated herpesvirus (KSHV) by the anthracyclines

**DOI:** 10.18632/oncotarget.2335

**Published:** 2014-08-10

**Authors:** Hyunju Kang, Jaehyung Song, Kwangman Choi, Hyeongki Kim, Miri Choi, So-Young Lee, Chonsaeng Kim, Sang Jun Lee, Moon Jung Song, Hyojeung Kang, Sung Hoon Back, Sang-Bae Han, Sungchan Cho

**Affiliations:** ^1^ Targeted Medicine Research Center, Korea Research Institute of Bioscience and Biotechnology, Cheongwon, Chungbuk, Republic of Korea; ^2^ Department of Biomolecular Science, University of Science and Technology, Daejeon, Republic of Korea; ^3^ College of Pharmacy, Kangwon National University, Chuncheon, Republic of Korea; ^4^ Department of Medical Biotechnology, Soonchunhyang University, Asan, Republic of Korea; ^5^ International Cooperation Office, Ministry of Food & Drug Safety, Cheongwon, Chungbuk, Republic of Korea; ^6^ Virus Research and Testing Group, Korea Research Institute of Chemical Technology, Daejeon, Republic of Korea; ^7^ Infection and Immunity Research Center, Korea Research Institute of Bioscience and Biotechnology, Daejeon, Korea; ^8^ Division of Biotechnology, College of Life Sciences and Biotechnology, Korea University, Seoul, Republic of Korea; ^9^ College of Pharmacy, Research Institute of Pharmaceutical Sciences, and Institute for Microorganisms, Kyungpook National University, Daegu, Republic of Korea; ^10^ School of Biological Sciences, University of Ulsan, Ulsan, Republic of Korea; ^11^ College of Pharmacy, Chungbuk National University, Cheongju, Chungbuk, Republic of Korea

**Keywords:** Kaposi's sarcoma-associated herpesvirus (KSHV), Lytic induction, Anthracyclines, Apoptosis, DNA intercalation

## Abstract

Lytic induction of latent Kaposi's sarcoma-associated herpesvirus (KSHV) has been considered as a therapeutic option for efficient treatment of several KSHV-associated malignancies. Here, we developed a robust high-throughput screening system that allows an easy and quantitative measurement of lytic induction of latent KSHV and discovered three anthracyclines as potent inducers from screen of FDA-approved drugs. Lytic induction of latent KSHV by three compounds was verified by the significant induction of lytic genes and subsequent production of infectious KSHV. Importantly, lytic induction by three compounds was much more efficient than that by sodium butyrate, a well-characterized inducer of KSHV lytic cycle. Mechanistically, the anthracyclines caused lytic induction of KSHV through apoptosis induced by their DNA intercalation rather than topoisomerase II inhibition. Consequently, our results clearly demonstrated a role of anthracyclines as effective lytic inducers of KSHV and also provided a molecular basis of their use for efficient treatment of diseases associated with KSHV infection.

## INTRODUCTION

Kaposi's sarcoma-associated herpes virus (KSHV), also known as human herpesvirus 8 (HHV-8), is a member of the gammaherpesvirus subfamily and is associated with several malignancies, such as Kaposi's sarcoma (KS), primary effusion lymphoma (PEL), and multicentric Castleman's disease [1, 2]. KSHV causes malignancies in individuals immunocompromised due to human immunodeficiency virus (HIV) infection or immunosuppressive drug therapies following transplantations. KS is the most common malignancy associated with acquired immune deficiency syndrome (AIDS). The majority of KSHV exists in a latent form in tumor cells, although a small population undergoes lytic replication. Even though highly-active antiretroviral therapies have dramatically reduced the incidence of KS in HIV-infected patients, KS remains the most common AIDS-associated malignancy in developed countries and is one of the most common cancers in developing countries.

Like other herpesviruses, KSHV exhibits two distinct life cycle phases after infection: lytic and latent replication. KSHV primarily establishes a lifelong latent infection in lymphocytes and endothelial cells, wherein the viral genome expresses only a subset of proteins, and its limited replication uses cellular machinery. Once the virus is reactivated from latency and enters the lytic cycle, most viral genes are expressed in a highly-ordered fashion (immediate-early, early, and late) [3], leading to production of infectious virions [4, 5].

The KSHV transition from the latent to lytic phase is tightly regulated by replication and transcription activator (RTA), a potent viral transactivator encoded by open reading frame (ORF) 50 [6]. RTA is necessary and sufficient to activate lytic replication in latently-infected cells [6-8]. RTA transcriptionally activates its own promoter and those of many lytic genes, including polyadenylated nuclear (PAN) RNA, ORF57, ORF21, and ORF36. In particular, the expression of PAN RNA is directly regulated by RTA. PAN RNA is the most abundant transcript among lytic genes and the regulatory elements in the PAN promoter are well-defined. Thus, PAN promoter activity has been prevalently used to easily assess lytic induction of KSHV [9].

Many antiviral drugs currently used to target KSHV are based on the inhibition of lytic replication [10]. For example, gancyclovir (GCV) is a nucleoside analog that is modified by viral proteins and eventually inhibits viral DNA polymerase activity [11, 12]. These drugs only target lytic KSHV and leave latent KSHV to be eradicated. Therefore, efficient lytic induction should be combined with lytic replication inhibition to fully treat KSHV-associated diseases. Lytic replication of KSHV can be induced in cultured cells by treatment with the phorbol ester, 12-0-tetradecanoyl phorbol 13-acetate, or calcium ionophores. These compounds induce RTA expression, subsequently triggering a cascade of lytic gene expression. However, their use in the clinical setting is hampered by severe side effects. Valproic acid, bortezomib, and prostratin induce lytic replication in KSHV-infected PEL cells [13-15]. Although these compounds have therapeutic potential, their effectiveness needs to be evaluated *in vivo*. Therefore, new therapeutic candidates need to be identified and evaluated *in vivo*. The identification of therapeutic candidate(s) from FDA-approved drugs is favorable because they have been proven safe in clinical settings, thus facilitating their therapeutic application to KSHV-associated diseases.

In this study, we developed a robust assay system based on luciferase as a reporter for quantitatively measuring lytic induction of KSHV. This system was used to screen approximately 650 FDA-approved drugs. Three anthracyclines were identified as potent and effective lytic inducers of KSHV by causing lytic induction in greater than 95% of cells. In contrast, sodium butyrate (SB) treatment resulted in lytic replication in only 5% of cells. Moreover, their unusual modes of action, RTA-independent activation of PAN promoter and apoptosis-mediated lytic induction, were found. Consequently, our results provided a molecular basis of the use of anthracyclines for KSHV-associated diseases.

## RESULTS

### Development of a high-throughput screening system for quantitatively measuring lytic induction of latent KSHV

**Figure 1:**
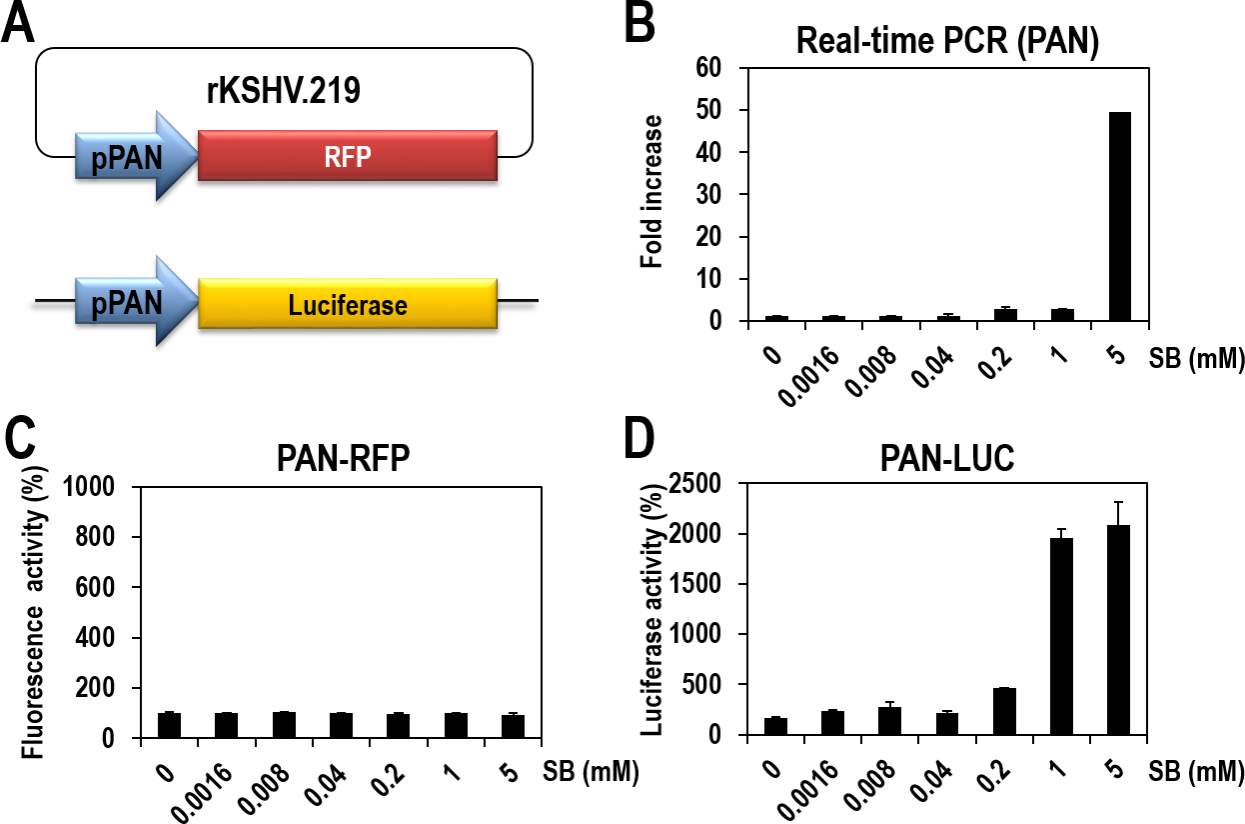
Establishment of vero-rKSHV.219/PAN-LUC cells for quantitatively measuring lytic induction of KSHV (A) Establishment of vero-rKSHV.219/PAN-LUC cells by introducing a PAN-LUC reporter into the original vero-rKSHV.219 cells. Using the PAN-LUC reporter, the expression of the firefly luciferase gene is controlled by the PAN promoter. (B) Vero-rKSHV.219 cells were treated with various concentrations of sodium butyrate (SB) for 24 hours. PAN transcripts were quantified by real-time PCR. The amount of PAN RNA in the DMSO-treated sample was set to one, and relative fold increases were calculated. Error bars represent standard deviations (SDs) from three independent experiments. (C) Vero-rKSHV.219 cells were treated with various concentrations of SB for 24 hours, and RFP signals were measured using a fluorescence reader. The RFP signal in the DMSO-treated sample was set to 100%, and relative fluorescence activities were calculated. Averages and SDs were determined from two independent experiments. (D) Vero-rKSHV.219/PAN-LUC cells were treated with various concentrations of SB for 24 hours. Firefly luciferase activity was measured using One-Glo reagents. Luciferase activity in the DMSO-treated sample was set to 100%, and relative luciferase activities were calculated. Averages and SDs were determined from two independent experiments.

The vero-rKSHV.219 cell line was previously established and has been prevalently used to investigate lytic induction of latent KSHV and to identify small-molecule inducers [16]. In this cell system, the expression of RFP is controlled by the PAN promoter. PAN is a representative lytic gene and its expression is highly responsive to RTA protein, thereby allowing for easy monitoring of lytic induction of latent KSHV. However, in our preliminary experiments with SB, a well-characterized lytic inducer, the RFP signal was too low and was restricted to only a small portion of cells for quantification. Therefore, we sought to develop a more robust assay system (vero-rKSHV.219/PAN-LUC) for easy and quantitative measurement of lytic induction of KSHV. We additionally introduced a PAN-LUC reporter, in which the luciferase gene is also expressed under the control of the PAN promoter, into the host genome of vero-rKSHV.219 cells (Figure 1A).

To evaluate the sensitivity of the new cells to lytic induction, the parental cells (vero-rKSHV.219) and the new cells (vero-rKSHV.219/PAN-LUC) were treated with SB in a dose-dependent manner. PAN RNA levels and the expression of both reporters (RFP and luciferase) were quantitatively measured for further comparison. In the parental cells, PAN RNA levels that were determined by real-time PCR analysis, gradually increased following SB treatment and were highest at 5 mM (Figure 1B), which is consistent with previous observations [16, 17]. On the contrary, the RFP signal, which reflects PAN promoter activity, was measured on a fluorescence reader and was barely responsive under the same conditions (Figure 1C). The discrepancy between RFP signals and actual levels of PAN RNA indicates that the RFP reporter system is not a sensitive measurement of lytic induction. Nevertheless, the RFP reporter system is valuable for assessing whether lytic induction occurs globally or in only a portion of cells by visualizing the lytic induction using fluorescence microscopy.

In contrast, the new cell system (vero-rKSHV.219/PAN-LUC) responded well to SB treatment, as judged by more than 20-fold increases in luciferase activity at 1 and 5 mM SB (Figure 1D). The highest activity of luciferase was observed at 1 mM SB, while the highest PAN RNA levels were detected at 5 mM SB by real-time PCR analysis. Therefore, the new cell system based on luciferase activity is more sensitive than the previous system (Figures 1B and 1D). Collectively, these results show that the PAN-LUC-based assay system is sensitive and suitable for high-throughput screening of lytic inducers.

### Identification of three anthracyclines as lytic inducers of KSHV

**Figure 2:**
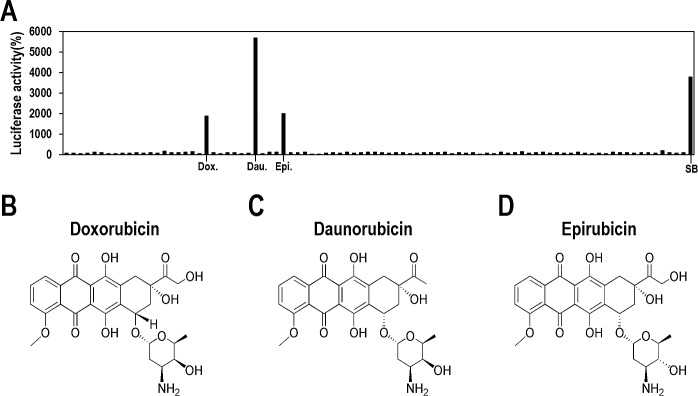
Screening FDA-approved drugs for induction of lytic induction of KSHV (A) Vero-rKSHV.219/PAN-LUC cells in 96-well plates were treated with approximately 650 FDA-approved drugs at 10 μM for 24 hours and assayed for firefly luciferase activity. SB (3 mM) was used as a positive control. A representative result from a plate containing the three compound hits is presented. (B, C, and D) The chemical structures of Doxorubicin, Daunorubicin, and Epirubicin, respectively.

Very few compounds have been reported to induce lytic replication of KSHV [13-15], and their clinical potentials need to be extensively evaluated. Therefore, we sought to identify new compound(s), particularly from previously FDA-approved drugs, with which clinical application for KSHV-associated diseases would be more feasible. To compare the effectiveness of lytic induction of KSHV, SB was included as a positive control. From screening of approximately 650 FDA-approved drugs, three compounds were identified that showed significant increases in luciferase activity, thus indicating lytic induction of KSHV (Figure 2A). Surprisingly, the compounds are all topoisomerase II inhibitors (Doxorubicin, Daunorubicin, and Epirubicin) and are structurally related to each other (Figures 2B, 2C, and 2D). They are classified as anthracyclines capable of intercalating DNA [18, 19], which majorly induce cytotoxic effect in cancer cells and enable them to be used to treat a wide range of cancers [20-22]. Of the three, Daunorubicin exhibited the highest luciferase activity, which was greater than that of SB (Figure 2A). Luciferase activities did increase following Doxorubicin or Epirubicin treatments, and they were comparable to that following SB treatment. These results indicate that some function of anthracyclines is associated with lytic induction of KSHV.

### Anthracyclines effectively induced lytic replication of KSHV

**Figure 3:**
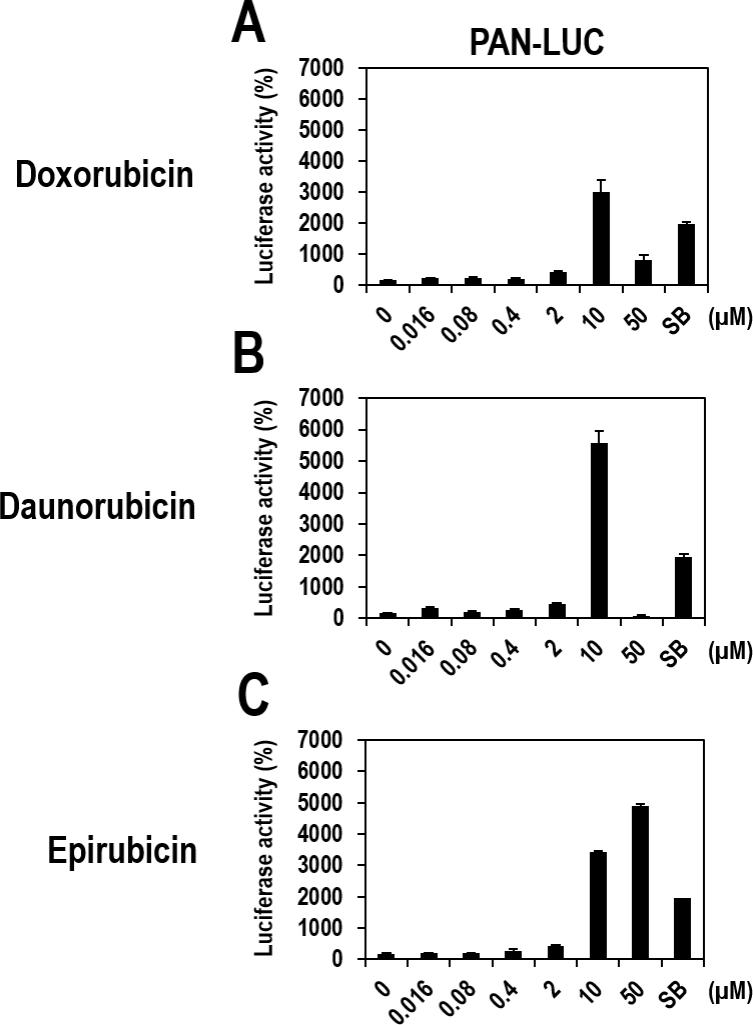
Dose-dependent activation of the PAN promoter by Doxorubicin, Daunorubicin, or Epirubicin in vero-rKSHV.219/PAN-LUC cells (A, B, and C) Vero-rKSHV.219/PAN-LUC cells were treated with increasing doses of Doxorubicin, Daunorubicin, or Epirubicin for 24 hours and then assayed for firefly luciferase activity. Effects of Doxorubicin, Daunorubicin, and Epirubicin on PAN promoter activity are separately presented in A, B, and C, respectively. SB (3 mM) was used as a positive control. The luciferase activity in the DMSO-treated sample was set to 100%, and relative luciferase activities were calculated. Averages and SDs were determined from two independent experiments.

To determine drug potency, vero-rKSHV.219/PAN-LUC cells were treated with various doses of the three compounds and SB as a positive control. All three compounds induced gradual increases in luciferase activity, with their highest activities (> 30-fold increases over the DMSO control) at 10 μM (Figure 3A, 3B, and 3C). The highest activities for each compound were greater than or comparable to that of SB. Epirubicin further induced luciferase activity at 50 μM, while luciferase activity induced by Doxorubicin or Daunorubicin decreased at the same concentration, which may have been caused by cell toxicity.

**Figure 4:**
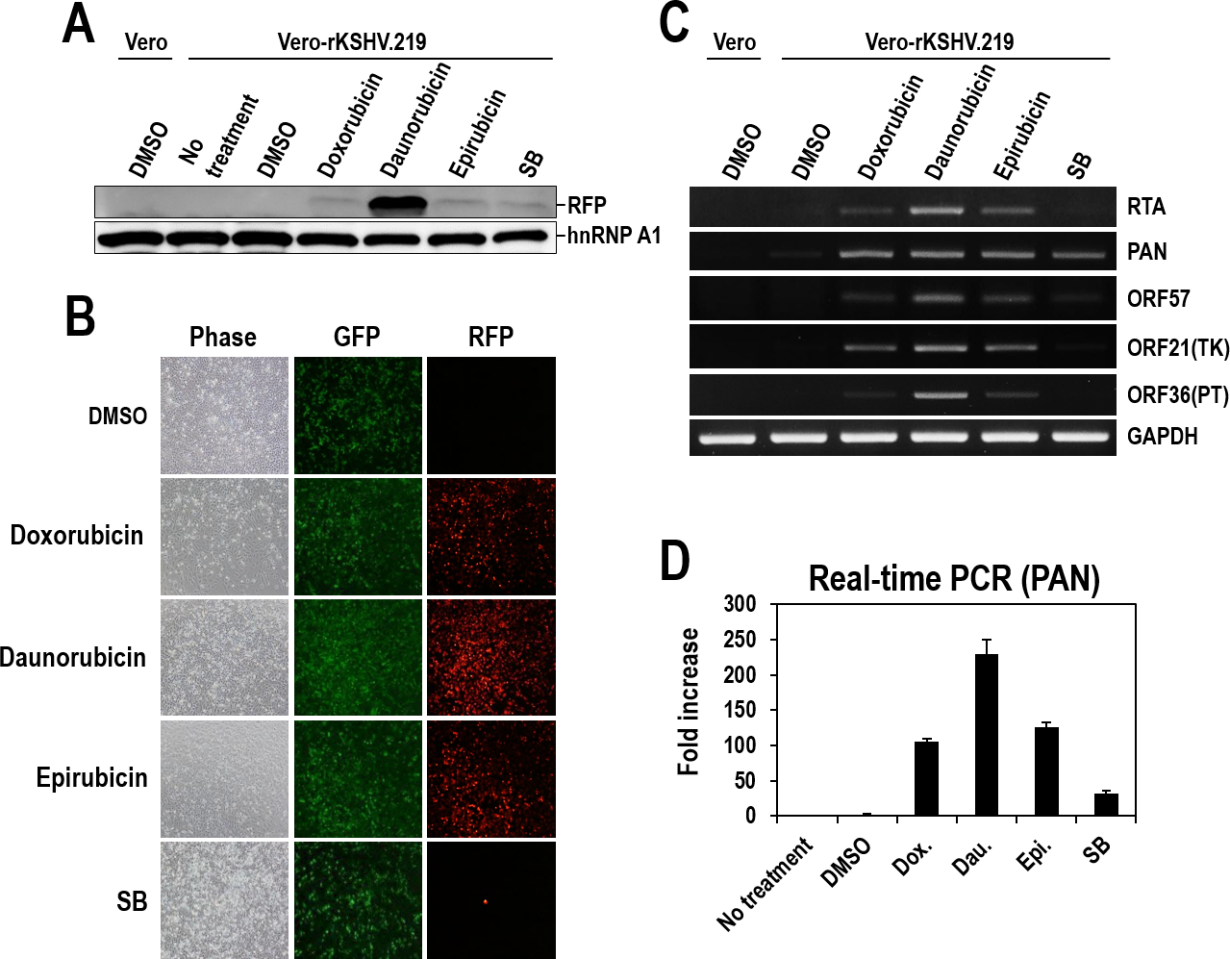
Validation of Doxorubicin, Daunorubicin, or Epirubicin as potent lytic inducers of KSHV (A) Vero-rKSHV.219 cells were treated with 10 μM Doxorubicin, Daunorubicin, or Epirubicin for 24 hours, and whole cell extracts were subjected to Western blot analysis with anti-RFP antibody. SB (3 mM) was used as a positive control. HnRNP A1 was analyzed as a loading control. (B) After incubation with 10 μM Doxorubicin, Daunorubicin, or Epirubicin for 24 hours, cells were visualized for GFP and RFP expression using fluorescence microscopy. Phase contrast images were also analyzed for morphological changes. (C) Total RNA was prepared from cells treated with 10 μM Doxorubicin, Daunorubicin, or Epirubicin and the expression of lytic genes (RTA, PAN, ORF57, ORF21, and ORF36) was analyzed using RT-PCR. GAPDH mRNA was analyzed as an internal control. (D) PAN RNA in compound-treated cells was quantitatively measured using real-time RT-PCR. The amount in the DMSO-treated sample was set to one, and fold increases were calculated. Averages and SDs were determined from two independent experiments.

To confirm that the three anthracyclines function as lytic inducers, the expression of another reporter, RFP, was analyzed by Western blotting and visualization of RFP signals. Consistent with the data from the PAN-LUC reporter, RFP expression was also detected in samples treated with the three individual compounds or SB. Daunorubicin induced the highest RFP expression (Figure 4A). The red fluorescence signal from RFP was also observed in all cells treated with the three individual compounds or SB. Unlike SB treatment that only induced RFP signals in a small portion of cells (<3%), treatment with any of the three compounds dramatically induced RFP signals in most of the cell population (>98%) (Figure 4B and Supplementary Figure 1). This observation that the three anthracyclines, newly identified as lytic inducers, affected the large majority of cells rather than only a limited number of cells is of great importance with regard to the effectiveness of lytic induction and complete eradication of latent KSHV via efficient lytic induction.

To further confirm lytic induction of KSHV, we examined the expression of other lytic genes in addition to PAN RNA. The three compounds efficiently induced the expression of all tested lytic genes (RTA, PAN, ORF57, ORF21, and ORF36) and SB only showed slight induction of each gene (Figure 4C). Consistent with the data in Figures 3 and 4A, Daunorubicin exhibited the highest induction of every examined lytic gene. The dramatic induction of PAN RNA following treatment with any of the three compounds was confirmed through real-time PCR analysis (Figure 4D).

### Anthracyclines produced infectious KSHV via lytic induction

**Figure 5:**
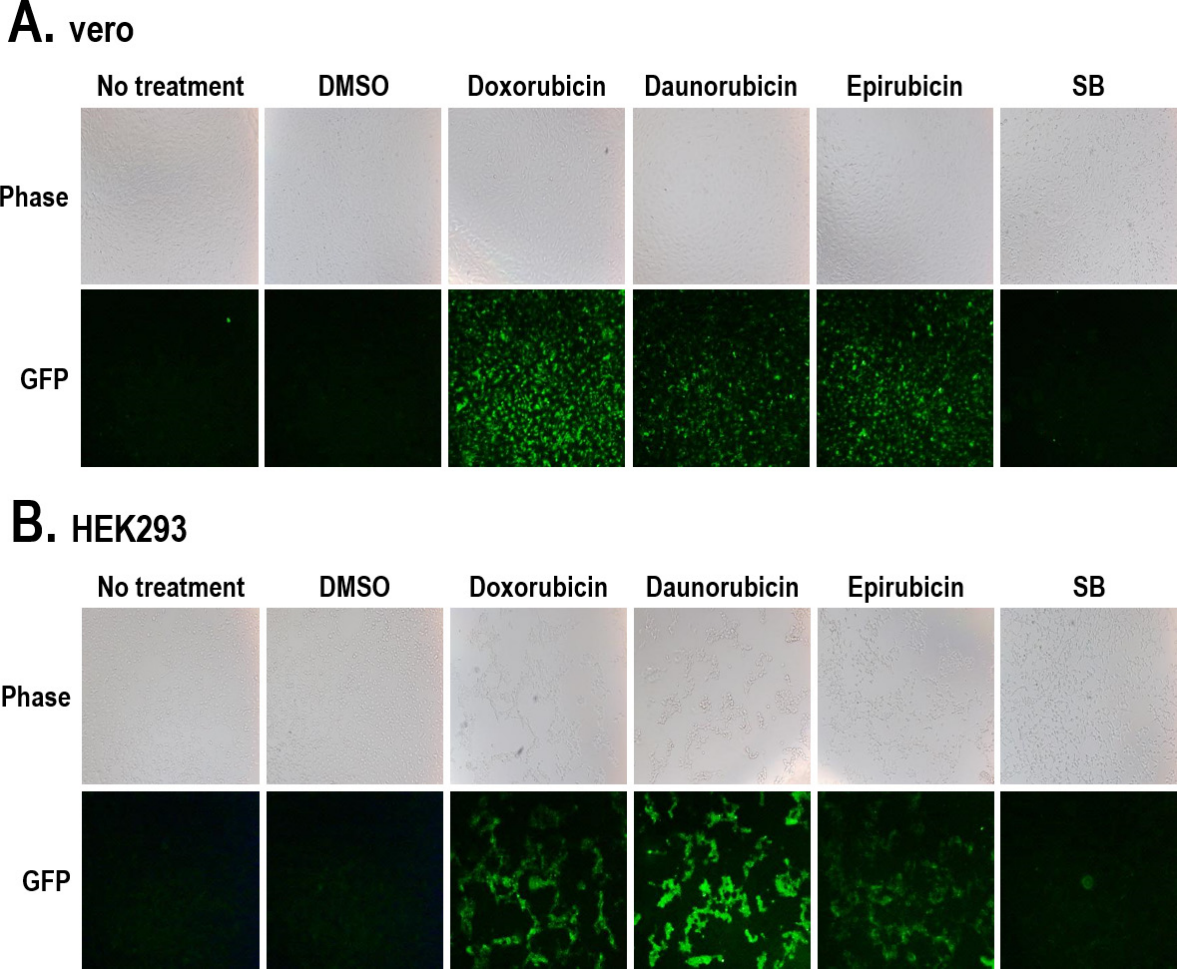
Production of rKSHV from vero-rKSHV.219 cells treated with the three compounds (A) Vero-rKSHV.219 cells were treated with 10 μM Doxorubicin, Daunorubicin, or Epirubicin for 24 hours, and culture media were harvested. Supernatants containing rKSHV were incubated with naïve vero (A) and HEK293 (B) cells for 48 hours. GFP signals were visualized using fluorescence microscopy. SB (3 mM) was used as a positive control.

Lytic induction accompanies a cascade of lytic gene expression and subsequent viral production followed by virus release and infection of other cells [23]. Therefore, we analyzed the late stage of the KSHV lytic cycle by harvesting cell media containing released KSHV for reinfecting naïve cells. Vero-rKSHV.219 cells were treated with the three individual compounds for 24 hours, and culture media were collected. Thereafter, naïve cells were incubated with these media for 48 hours, and the newly-induced GFP signals were analyzed. The rKSHV.219 genome contains the GFP gene under the control of the mammalian elongation factor 1a (EF-1a) promoter, which allows for efficient expression of GFP in host cells after rKSHV.219 infection. The detection of GFP signals in naïve cells after incubation with cell media indicates the production and release of infectious virus from parental cells (vero-rKSHV.219). Naïve vero cells exhibited strong GFP signals after incubation with culture media from cells treated with the three compounds (Figure 5A). A similar pattern was also observed in human HEK293 cells incubated with identical cell media (Figure 5B). Compared to the GFP signals induced by the three compounds, those induced by SB were extremely low and barely detectable. These results indicate that the three compounds induced lytic replication accompanying lytic gene expression and subsequent production of infectious KSHV in vero-rKSHV.219 cells.

### Anthracyclines induced lytic gene expression in human B-cell lymphoma BCBL1 cells naturally infected with KSHV

**Figure 6:**
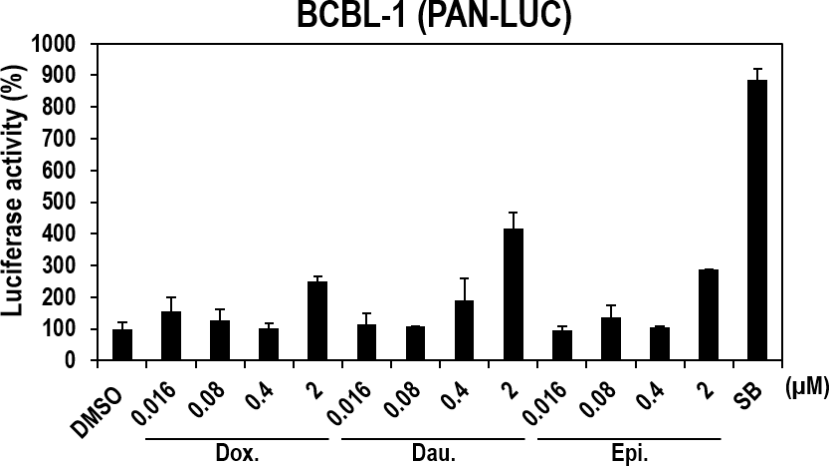
Effects of the three compounds on PAN promoter activities in human B-cell lymphoma BCBL1 cells naturally infected with KSHV Human BCBL1 cells naturally infected with KSHV were transfected with plasmids expressing firefly luciferase under the control of the PAN promoter. At 12 hours post-transfection, cells were treated with increasing doses of Doxorubicin, Daunorubicin, or Epirubicin for 18 hours and assayed for firefly luciferase activity. SB (3 mM) was used as a positive control. The luciferase activities in the DMSO-treated samples were set to 100%, and relative activities were calculated. Averages and SDs were determined from two independent experiments.

Lytic induction in response to the three anthracyclines was demonstrated in nonhuman vero cells. We examined the lytic-inducing effect of the three compounds in human B-cell lymphoma (BCBL-1) cells that were naturally infected with KSHV. To optimize the conditions of compound treatment, the lytic-inducing effect of Daunorubicin, which consistently showed the highest activities in every experiment, was first investigated. BCBL1 cells were transfected with plasmid expressing luciferase under the control of the PAN promoter and treated with Daunorubicin for various times (6, 12, and 18 hours). As a result, incubation with Daunorubicin at 0.4 and 2 μM for 18 hours induced the highest luciferase activity (Supplementary Figure 2). Therefore, the other compounds were also tested under these conditions in the following experiments. As expected, all three compounds significantly induced the luciferase activity of the PAN promoter more than 2-fold, particularly at 2 μM (Figure 6), in spite of slight decreases in cell viability (Supplementary Figure 3). Consistent with the data in vero cells (Figure 3 and 4), the treatment of Daunorubicin also exhibited the highest luciferase activity in BCBL1 cells, even though its activity was lower than that of SB. Therefore, the three compounds induced lytic gene expression in human B-cell lymphoma naturally infected with KSHV as they did in vero cells.

### Anthracyclines induced expression of lytic genes independently of viral proteins

**Figure 7:**
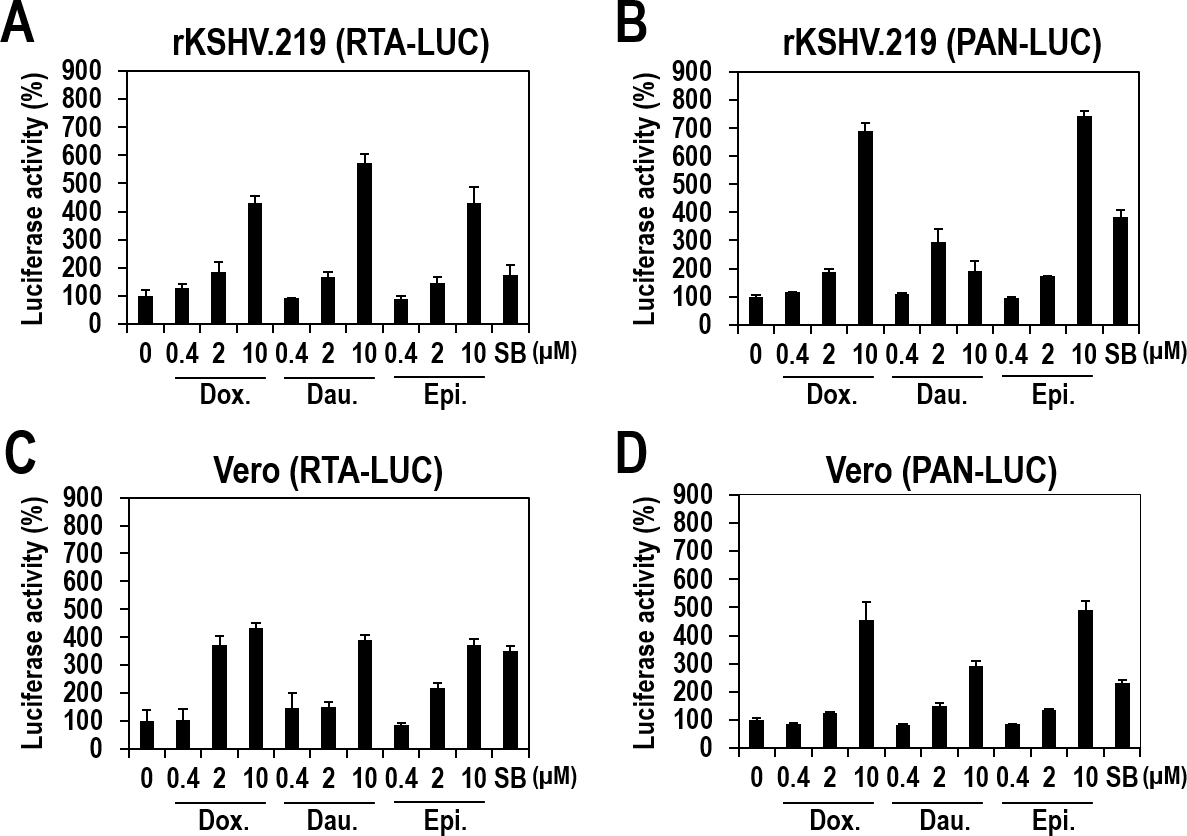
KSHV-independent activation of the RTA and PAN promoters by the three compounds (A and B) Vero-rKSHV.219 cells were transfected with plasmids expressing firefly luciferase under the control of the RTA (A) or PAN (B) promoters At 24 hours post-transfection, cells were treated with increasing doses of Doxorubicin, Daunorubicin, or Epirubicin for 24 hours and assayed for firefly luciferase activity. SB (3 mM) was used as a positive control. The luciferase activities in the DMSO-treated samples were set to 100%, and relative activities were calculated. Averages and SDs were determined from two independent experiments. (C and D) Vero cells were transfected with plasmids expressing firefly luciferase under the control of the RTA (C) or PAN (D) promoters. At 24 hours post-transfection, cells were treated with increasing doses of Doxorubicin, Daunorubicin, or Epirubicin for 24 hours and assayed for firefly luciferase activity.

Many compounds that induce lytic replication upregulate expression of lytic genes in an RTA-dependent manner [24] by upregulating RTA expression that then induces a cascade of downstream gene activation. To determine if the three topoisomerase II inhibitors also function in this manner, vero-rKSHV.219 cells were transfected with plasmids expressing luciferase under the control of PAN or RTA promoters, and treated with the three compounds. All three compounds induced the PAN promoter-controlled luciferase activity by more than 3-fold (Figure 7B). Similarly, the luciferase activity controlled by the RTA promoter was enhanced by more than 4-fold (Figure 7A). These data suggest that the three compounds likely induce PAN promoter activity in an RTA-dependent manner. However, further experiments redirected our preliminary conclusion.

To determine whether viral proteins are involved in lytic induction in response to the three compounds, we performed the same experiments with vero cells. Uninfected vero cells were transfected with plasmids expressing luciferase under the control of the PAN or RTA promoters and were treated with the three compounds. Strikingly, all three compounds induced luciferase activity of both PAN and RTA promoters to similar extents (Figure 7C and 7D), indicating that activation of the PAN promoter is actually independent of RTA activation. Consequently, the three compounds functioned on both promoters separately. Moreover, the promoter activation was not dependent on viral protein(s) because there were no viral proteins in uninfected vero cells. Viral protein-independent activation of the PAN promoter was also confirmed by the significant increase in luciferase activity in BJAB cells, a human B-cell lymphoma line without KSHV infection [25, 26], following treatment with the three compounds (Supplementary Figure 4). Collectively, these results demonstrated that the three anthracycliness regulate the PAN and RTA promoters individually and their activation is not dependent on any viral proteins as effectors.

### Lytic induction of KSHV by anthracyclines is not dependent on topoisomerase II inhibition

**Figure 8:**
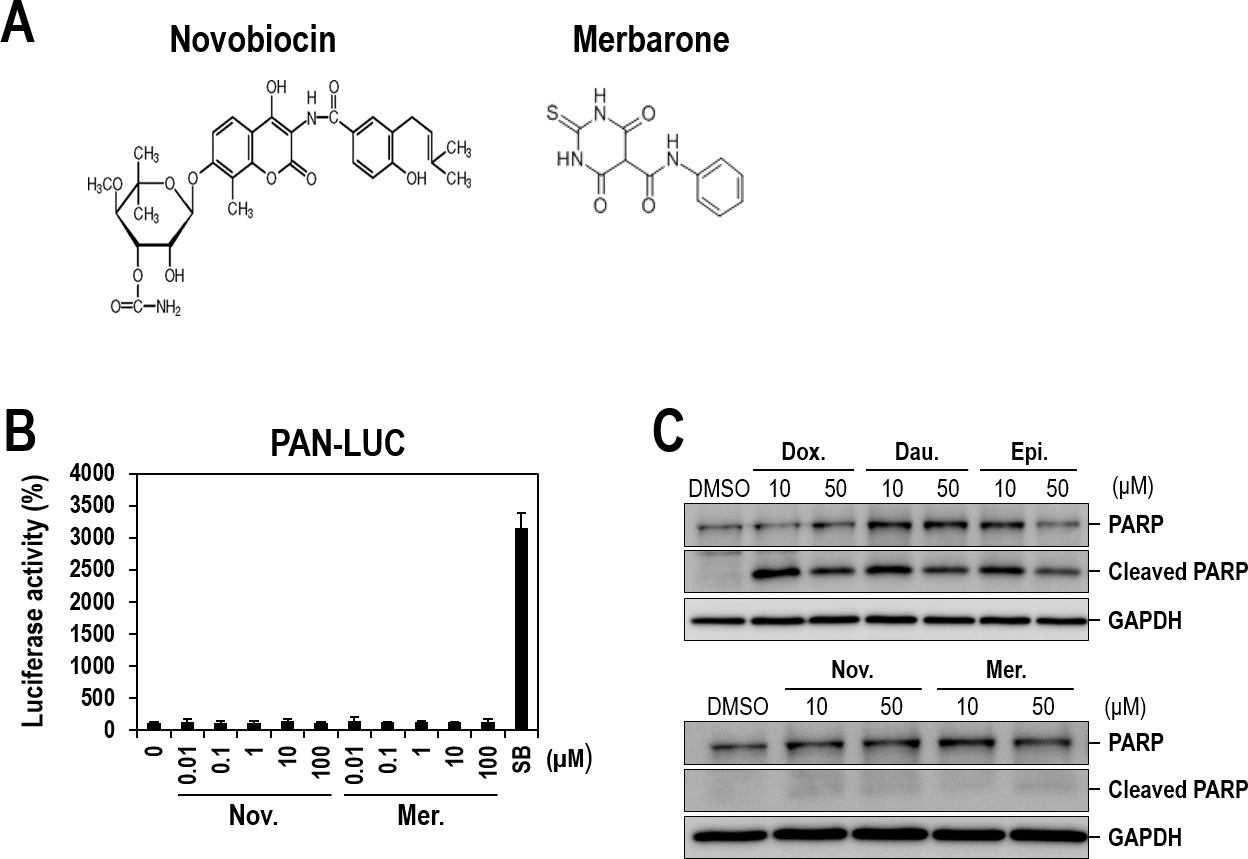
Lytic induction of KSHV by anthracycline-induced apoptosis (A) The chemical structures of Novobiocin and Merbarone. (B) Vero-rKSHV.219/PAN-LUC cells were treated with increasing doses of topoisomerase II catalytic inhibitors (Novobiocin and Merbarone) for 24 hours and assayed for firefly luciferase activity. SB (3 mM) was used as a positive control. The luciferase activity in the DMSO-treated sample was set to 100%, and relative activities were calculated. Averages and SDs were determined from two independent experiments. (C) PARP cleavage induced by three anthracyclines. Vero-rKSHV.219/PAN-LUC cells were treated with 10 and 50 μM of Doxorubicin, Daunorubicin, Epirubicin, Novobiocin or Merbarone for 24 hours, and whole cell extracts were subjected to Western blot analysis with anti-PARP antibody. GAPDH was analyzed as a loading control.

Anthracyclines have several mechanisms of action such as topoisomerase II inhibition, DNA intercalation, generation of free radicals, and induction of histone eviction from chromatin [18, 19]. In order to determine whether topoisomerase II inhibition is crucial for lytic induction of KSHV, we tested additional topoisomerase II inhibitors that are structurally different from anthracyclines and do not intercalate DNA. Particularly, we included two known topoisomerase II catalytic inhibitors (Novobiocin and Merbarone) (Figure 8A). Surprisingly, topoisomerase II inhibitors other than three anthracyclines had no effect on lytic induction of KSHV even at the highest concentrations (100 or 50 μM) (Figure 8B or Supplementary Figure 5, respectively). Note that IC_50s_ of Novobiocin and Merbarone for topoisomerase II were consistently reported as 10-20 μM [27]. These results suggest that anthracyclines have an effect on lytic induction of KSHV most probably through DNA intercalation rather than topoisomerase II inhibition.

### Lytic induction of KSHV by anthracyclines-induced apoptosis

Recently, it has been proposed that KSHV lytic activation is alternatively initiated through apoptosis [28]. In this study, cytotoxic chemotherapeutic agents including doxorubicin were demonstrated to induce lytic replication of KSHV. These previous report in conjuction with our observation suggest that only DNA-intercalating anthracyclines out of various topoisomerase II inhibitors might activate lytic replication of KSHV through inducing apoptosis. On the contrary, other topoisomerase II inhibitors which are not capable of intercalating DNA might not induce apoptosis as sufficiently as to attain KSHV activation. In order to prove this hypothesis, we examined the involvement of apoptosis in anthracyclines-induced lytic induction of KSHV. In order to correlate lytic-inducing capability of anthracyclines with apoptosis, apoptosis was simply analyzed by detecting PARP cleavage, which is a representative event during this process [29]. As expected, only three anthracyclines exhibited an obvious cleavage of PARP, while other topoisomerase II inhibitors had no effect (Figure 8C). These results suggest that three anthracyclines including doxorubicin induced lytic replication of KSHV through inducing apoptosis.

## DISCUSSION

Latent infection of KSHV makes it difficult to achieve complete eradication of KSHV with the currently available antiviral drugs. This is why lytic induction of latent KSHV combined with known antiviral drugs is considered an efficient therapeutic option for treatment of KSHV-associated diseases. However, very few compounds have been identified as lytic inducers and their clinical potential needs to be evaluated further. More importantly, their effectivenesss for lytic induction of KSHV is too poor to achieve complete eradication of KSHV from infected cells. In our study, we identified three anthracyclines (Doxorubicin, Daunorubicin, and Epirubicin) as unprecedently effective lytic inducers from a screen of approximately 650 FDA-approved drugs.

We first developed a robust assay system that quantitatively measures lytic induction of KSHV in vero-rKSHV.219 cells that have been prevalently used by many researchers [16, 30, 31]. In our preliminary experiments, the original vero-rKSHV.219 cells containing the RFP gene under the control of the PAN promoter were not sensitive enough to be used for high-throughput screening. Therefore, we introduced a highly-sensitive reporter luciferase (PAN-LUC) whose expression is controlled by the PAN promoter into vero-rKSHV.219 cells. Greatly improved sensitivity was demonstrated by the use of sodium butyrate (SB) as a representative lytic inducer of KSHV (Figure 1). This upgraded system (vero-rKSHV.219/PAN-LUC) allowed for high-throughput screening with high sensitivity and analysis of the effectiveness of candidate compounds at a single-cell level by visualizing the RFP signal.

Several lines of evidence demonstrated that the three anthracyclines are potent and effective lytic inducers of KSHV in nonhuman cells (vero) and human B-cell lymphoma cells (BCBL1) naturally infected with KSHV. First, the three compounds dose-dependently induced the expression of luciferase under the control of the PAN or RTA promoters in BCBL1 and vero cells (Figure 6 and 3, respectively). Second, their lytic-inducing activities were confirmed by increases in RFP expression, another reporter in vero-rKSHV.219 cells (Figures 4A and 4B). Third, the three compounds dramatically induced expression of lytic genes (RTA, PAN, ORF57, ORF21, and ORF36) in vero-rKSHV.219 cells as determined by RT-PCR (Figures 4C and 4D). Fourth, all three compounds efficiently produced infectious viruses from vero-rKSHV.219 cells that were judged by the GFP signal in newly-infected vero and HEK293 cells (Figure 5).

The effectiveness of anthracyclines as lytic inducers was consistently higher than or comparable to that of SB throughout most of the experiments (Figures 2, 3, 4, and 5). More importantly, lytic induction induced by the three compounds was observed in more than 95% of the cells, while SB induced lytic replication in less than 5% of the cells as assessed by RFP signals in vero-rKSHV.219 cells (Figure 4B). Similarly, the effectiveness of the three compounds was observed by using viruses produced following treatment with the compounds for reinfection. The titer of viruses produced following treatment with the three compounds was much higher than that produced by SB (Figure 5). Collectively, these results demonstrate that anthracyclines are much more effective drugs for achieving efficient lytic induction of KSHV than is SB.

How do anthracyclines induce lytic replication? Many lytic-inducing compounds, including SB, induce RTA expression that subsequently triggers a cascade of lytic gene expression. Unlike these compounds, the three anthracyclines acted on both of the PAN and RTA promoters individually rather than by following a cascade of lytic gene activation (Figure 7). Moreover, lytic induction by the three compounds is not dependent on any viral proteins (Figure 7). The study with other topoisomerase II inhibitors revealed that lytic induction by anthracyclines is not related to topoisomerase II inhibition (Figure 8B and Supplementary Figure 5). Rather, DNA intercalating activity which only anthracyclines have is likely to be crucial. Anthracyclines are capable of intercalating into DNA base pairs and cause inhibition of DNA replication, thus allowing them to work as chemotherapeutic drugs for a wide range of cancers [19, 32]. Nevertheless, we cannot completely exclude the possibility that other functions of anthracyclines such as generation of free radicals and induction of histone eviction from chromatin might be contributing to lytic induction of KSHV. Intriguingly, it has been recently proposed that KSHV activation is induced by apoptosis [28]. In this study, doxorubicin, one of anthracyclines, was demonstrated to induce KSHV activation by inducing apoptosis. Our studies extended this previous observation by showing that anthracyclines in addition to doxorubicin commonly induce lytic replication of KSHV through apoptosis, particularly, induced by their DNA intercalation rather than topoisomerase II inhibition (Figure 8). Still, it remains unclear how apoptosis activates the transcription of PAN/RTA genes. Further investigation will clarify this issue.

Topoisomerase II inhibitors have been recently reported to be inhibitors of lytic and latent KSHV replication [27, 33]. Our finding demonstrates another effect of anthracyclines, a class of topoisomerase II inhibitors, as potent and effective lytic inducers of KSHV. Even though anthracyclines have not been tested so far, it is conceivable that they will be functioning as inhibitors of lytic and latent KSHV replication as other topoisomerase II inhibitors. Since a single compound can function as an efficient inducer of the lytic cycle, as well as an inhibitor of lytic and latent replication, anthracyclines could be considered an ideal class of drug for treatment of KSHV-associated diseases. Anthracyclines have already been FDA-approved as chemotherapy drugs for various cancers such as hematological malignancies, many types of carcinoma, and soft tissue sarcomas (30, 31). Doxorubicin, one of anthracyclines identified here, is currently being used as a therapy even for Kaposi's sarcoma [34-36]. Regardless of the multiple actions of anthracyclines on the KSHV life cycle, a combination of these anthracyclines with known antiviral drugs, such as GCV, is worth consideration as another option for the effective treatment of KSHV-associated diseases. Likewise, Epstein-Barr virus (EBV), another virus in the gammaherpesvirus subfamily, is reactivated following treatment with Doxorubicin [11], suggesting that anthracyclines may have broad potential treatment of gammaherpesvirus subfamily-related diseases. The evaluation of the therapeutic potential of these anthracyclines in an MHV68-infected animal model will be pursued in future studies.

Here, we identified three anthracyclines as potent and effective lytic inducers of KSHV. These FDA-approved drugs will provide various therapeutic options for KSHV-associated diseases.

## MATERIALS AND METHODS

### Cell culture

African green monkey kidney cells (vero), vero-rKSHV.219 cells, and human embryonic kidney 293 cells (HEK293) were maintained in DMEM medium containing 10% FBS (Hyclone) and 1% penicillin-streptomycin. Vero-rKSHV.219 cells were kindly provided by Dr. Jae U. Jung (University of Southern California). Primary effusion lymphoma cells (BCBL-1) naturally-infected with KSHV and B-cell lymphoma cells (BJAB) were grown in RPMI 1640 medium (Welgene) supplemented with 10% FBS (Welgene) and 1% penicillin-streptomycin.

### Establishment of the vero-rKSHV.219/PAN-LUC cell line

To construct the pGL4.14-PAN-LUC reporter plasmid, DNA spanning nt -122 to +14 of the PAN promoter [37] was obtained from vero-rKSHV.219 cells using RT-PCR with appropriate primers (forward, 5'-TGGCCTAACTGGCCGGTACCAGGGT CAGCTTGAAGGATGAT-3' and reverse, 5'-CCGGATTGCCAAGCTTTGGGCAGTCCC AGTGCTAAAC-3') and inserted into Kpn I/Hind III restriction sites of pGL4.14 (Promega). Plasmids were transfected into vero-rKSHV.219 cells using X-tremeGENE siRNA Transfection Reagent (Roche), and transfectants were selected using puromycin (1 μg/mL) for more than 1 week. Selected colonies were individually amplified and treated with 3 mM SB for 24 hours. Cells (vero-rKSHV.219/PAN-LUC) showing the highest responsiveness to SB were chosen and maintained for further experiments.

### High-throughput screening of FDA-approved drugs

An FDA-approved drugs library was purchased from Selleckchem (http://www.selleckchem.com). To screen approximately 650 FDA-approved drugs, vero-rKSHV.219/PAN-LUC cells (2 × 10^4^ cells/well) were seeded in each well of 96-well plates and incubated with 10 μM individual drugs for 24 hours. Firefly luciferase activity was measured using a One-Glo Luciferase Assay System (Promega) as per the manufacturer's protocol.

### Luciferase assays for lytic induction of KSHV

To measure induction of the RTA and PAN promoters in response to the active compounds from the high-throughput screen, vero and vero-rKSHV.219 cells (3 × 10^5^ cells/well) in 6-well plates were transfected with 2 μg of the reporter plasmids pGL3-Rp [38] and pGL4.14-PAN-LUC using X-tremeGENE DNA transfection reagent. Twenty-four hours post-transfection, cells were incubated with various concentrations of Doxorubicin, Daunorubicin, or Epirubicin for 24 hours and assayed for firefly luciferase activity. BCBL-1 cells (2 × 10^6^ cells/well) were seeded in 6-well plates 3 hours prior to transfection with 4 μg of reporter plasmid using Lipofectamine 2000 (Promega). Twelve hours post-transfection, various concentrations of Doxorubicin, Daunorubicin, or Epirubicin were added for 18 hours, and firefly luciferase activities were measured. Cell viability was also measured using CellTiter-Glo Luminescent Cell Viability Assays (Promega) as per the manufacturer's protocol.

### Analysis of red fluorescence protein (RFP) signals in vero-rKSHV.219 cells

Vero-rKSHV.219 cells incubated with Doxorubicin, Daunorubicin, or Epirubicin for 24 hours were washed with phosphate-buffered saline (PBS) and fixed with 4% paraformaldehyde at room temperature. Fixed cells were washed with PBS twice and RFP signals were visualized using fluorescence microscopy.

### Quantitative Western blot analysis

Quantitative Western blot analyses were performed as previously described [39]. Briefly, whole-cell extracts were resolved by SDS-PAGE and transferred to PVDF membranes (GE Healthcare). Membranes were blocked in 5% skim milk and incubated overnight at 4°C. Proteins that reacted with antibodies were detected on membranes using a WEST-ZOL Plus Western blotting detection system (Intron Biotechnology) and analyzed with an LAS-4000 image analyzer (Fujifilm, Tokyo, Japan). Anti-RFP, -GAPDH, and -PARP antibodies were purchased from MBL International, Sigma, and Abcam, respectively. Anti-hnRNP A1 antibody was kindly provided by Gideon Dreyfuss (University of Pennsylvania).

### RT-PCR and quantitative real-time PCR

To examine the induction of lytic genes, vero-rKSHV.219 cells were treated with Doxorubicin, Daunorubicin, or Epirubicin for 24 hours. Total RNA was extracted using TRIzol reagent (Invitrogen) followed by cDNA synthesis using Omniscript RT kits (Qiagen) and oligo-dT primers. PCR was performed using GoTaq Green Master Mix (Promega). The primer sequences used for PCR were as follows: RTA (forward, 5'-GTGCATTCGGATTATGAAAGAA-3' and reverse, 5'-TACGTGTTGTAGAGCTTCAGT-3'), PAN (forward, 5'-TTATGATATGCGAGGATACTTAA -3' and reverse, 5'-TGTCATTCAAATCGACTTGCTT-3'), ORF57 (forward, 5'-ATAAGAGCAGGGGGCAAAGA-3' and reverse, 5'-TGACCACCTCCACAGACAGATG-3'), ORF21 (forward, 5'-GATCATGTTGCAGTACATCAC-3' and reverse, 5'-TGTCTCCTCTAGGTGATTAAC-3'), ORF36 (forward, 5'-ACCATCGACAT GTCCTCGTT-3' and reverse, 5'-AAGTCTCGCTCTAG TAGCTTT-3'), and GAPDH (forward, 5'-GAACGGGAAGCTT GTCATCAATGG -3' and reverse, 5'-TGTGGTCATGAGTCCTTCCACGAT-3'). Quantitative real-time PCR of PAN transcripts was performed using SYBR Green PCR 2 × Master Mix (Bio-Rad) with the same PAN primers.

### Reinfection of vero and HEK293 cells with compound-treated vero-rKSHV.219 cell media

Vero-rKSHV.219 cells were seeded in 6-well plates one day prior to treatment with the following compounds. Cells were treated with 10 μM Doxorubicin, Daunorubicin, or Epirubicin for 24 hours, and culture media were harvested. To remove cell debris, culture media were subjected to high-speed centrifugation at 16,000 × g, and only supernatants were used for the reinfection experiment. HEK293 and vero cells were incubated with these supernatants for 48 hours, and newly-induced GFP signals were analyzed by fluorescence microscopy.

## SUPPLEMENTARY MATERIAL AND FIGURES


