# Supplementary material for: Efficient lytic induction of kaposi's sarcoma-associated herpesvirus (KSHV) by the anthracyclines
Source: Oncotarget. 2014 Aug 10;5(18):8515–27. doi: 10.18632/oncotarget.2335 (PMC4226701; doi:10.18632/oncotarget.2335)
Supplement: Supplementary file 1 [file oncotarget-05-8515-s001.pdf]

Efficient lytic induction of kaposi's sarcoma-associated herpesvirus (KSHV) by the anthracyclines

Supplementary Material

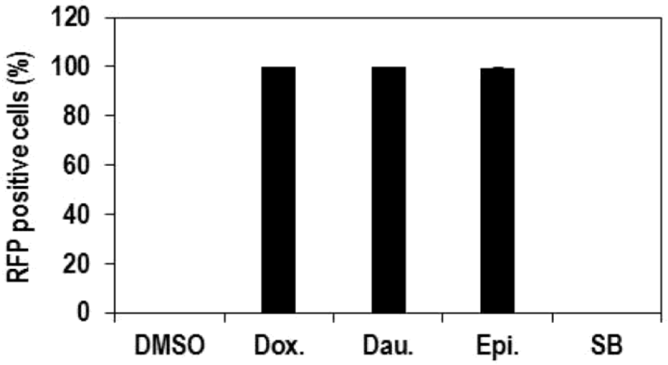

Supplementary Figure 1: Lytic induction by three anthracyclines occurred in most of cell populations

After incubation with 10 μM Doxorubicin, Daunorubicin, or Epirubicin for 24 hours, cells were visualized for RFP expression using fluorescence microscopy. RFP positive cells for each were counted and relative ratios in percentage were calculated. Averages and SDs were determined from two independent experiments.

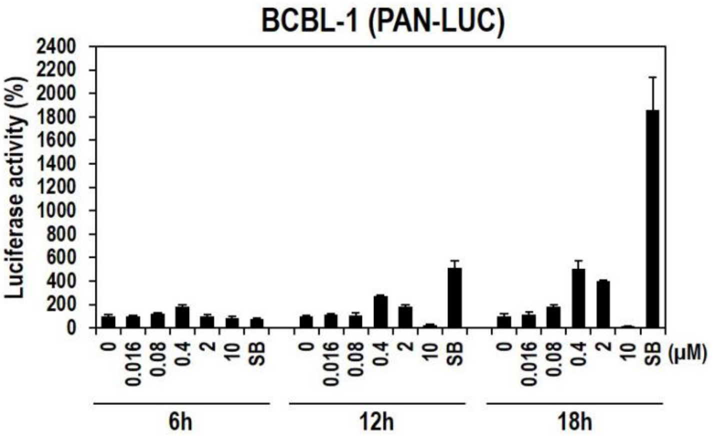

Supplementary Figure 2: Effect of Daunorubicin on PAN promoter activity in BCBL1 cells Human BCBL1 cells naturally infected with KSHV were transfected with plasmid expressing firefly luciferase under the control of the PAN promoter. At 12 hours post-transfection, cells were treated with increasing doses of Daunorubicin for various times (6, 12, and 18 hours) and assayed for firefly luciferase activity. SB (3 mM) was used as a positive control. The luciferase activities in the DMSO-treated samples for each time were set to 100%, and relative activities were calculated. Averages and SDs were determined from two independent experiments.

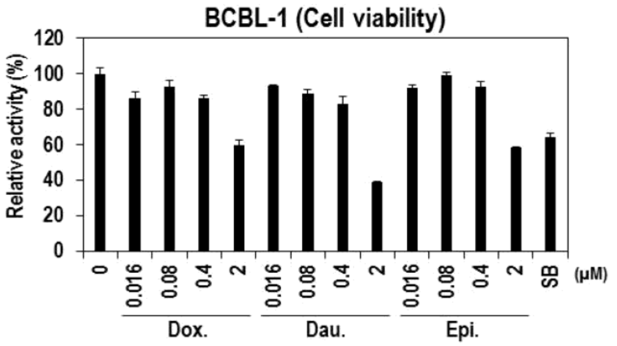

Supplementary Figure 3: Decreased viability of BCBL1 cells by three anthracyclines Human BCBL1 cells were treated with increasing doses of Doxorubicin, Daunorubicin, or Epirubicin for 18 hours, and assayed for cell viability using CellTiter-Glo reagent. SB (3 mM) was used as a positive control. The activity in the DMSO-treated sample was set to 100%, and relative luciferase activities were calculated. Averages and SDs were determined from two independent experiments.

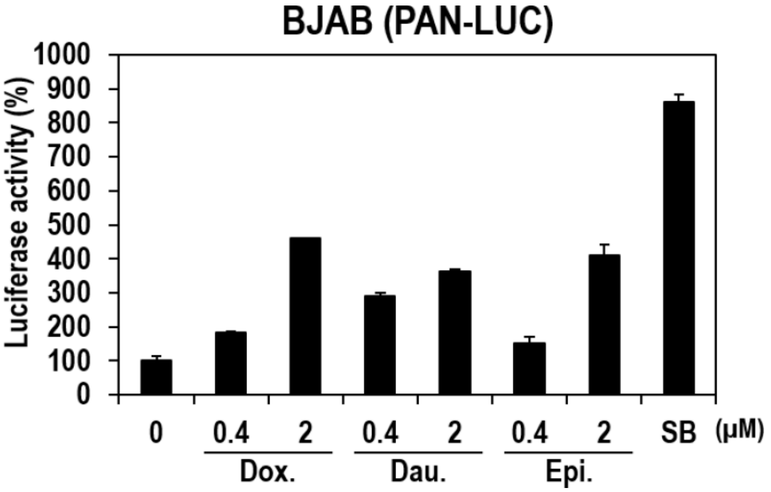

Supplementary Figure 4: Effects of the three compounds on PAN promoter activity in BJAB cells Human B-cell lymphoma BJAB cells without KSHV were transfected with plasmids expressing firefly luciferase under the control of the PAN promoter. At 12 hours post-transfection, cells were treated with increasing doses of Doxorubicin, Daunorubicin, and Epirubicin for 18 hours and assayed for firefly luciferase activity. SB (3 mM) was used as a positive control. The luciferase activity in the DMSO-treated sample was set to 100%, and relative activities were calculated. Averages and SDs were determined from two independent experiments.

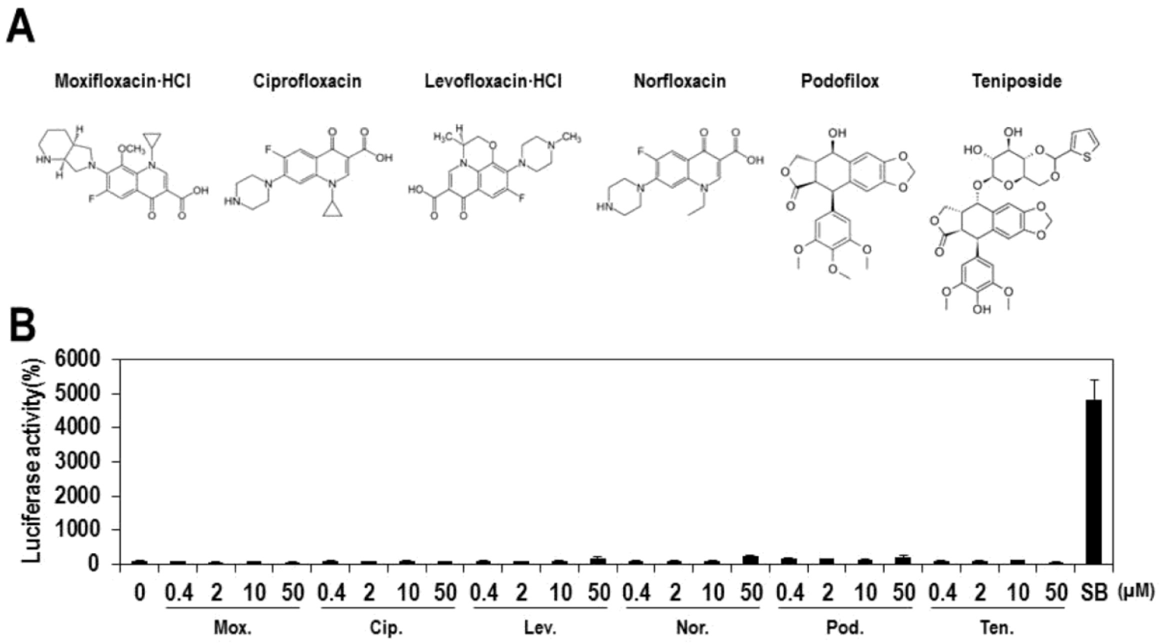

Supplementary Figure 5: Effects of topoisomerase II inhibitors on PAN promoter activity in vero-rKSHV.219/PAN-LUC cells Vero-rKSHV.219/PAN-LUC cells were treated with increasing doses of topoisomerase II inhibitors for 24 hours and assayed for firefly luciferase activity. SB (3 mM) was used as a positive control. The luciferase activity in the DMSO-treated sample was set to 100%, and relative activities were calculated. Averages and SDs were determined from two independent experiments.
